# Frequency of Regulatory T-Cells in the Peripheral Blood of Patients with Pulmonary Tuberculosis from Shanxi Province, China

**DOI:** 10.1371/journal.pone.0065496

**Published:** 2013-06-06

**Authors:** Hui Pang, Qin Yu, Buping Guo, Yi Jiang, Li Wan, Jun Li, Yanjun Wu, Kanglin Wan

**Affiliations:** 1 Department of Immunology, Changzhi Medical College, Changzhi, Shanxi Province, P. R. China; 2 National Institute for Communicable Disease Control and Prevention, Chinese Center for Disease Control and Prevention/State Key Laboratory for Infectious Disease Prevention and Control, Beijing, P. R. China; 3 Department of Laboratory Medicine, Changzhi Medical College, Shanxi Province, P. R. China; 4 Pathogenic Biology Institute, University of South China, Hengyang, P. R. China; 5 The People's Hospital of Changzhi City, Shanxi Province, P. R. China; The Ohio State University, United States of America

## Abstract

**Background:**

Tuberculosis (TB) is a disease caused by the chronic and continuous infection of the pathogen *Mycobacterium tuberculosis* (*M. tuberculosis*). *M. tuberculosis* is an intracellular bacterial pathogen and is eliminated mainly through CD4^+^ effector Th cells. *M. tuberculosis* induces regulatory T lymphocytes (Tregs) that mediate immune suppression by cell-to-cell contact or by secreting cytokines such as transforming growth factor-β (TGF-β). To understand the role of regulatory T-cells in the pathogenesis of TB, we have measured the in vivo frequency of regulatory T-cells and associated in vivo cytokine production in pulmonary tuberculosis patients.

**Methodology/Principal Findings:**

In this study, we analyzed blood samples from 3 different populations (Group 1: patients with active TB, Group 2: patients recovered from TB and Group 3: healthy controls). We measured natural regulatory T-cell expression in peripheral blood using flow cytometry, and levels of blood serum IFN-γ and TGF-β1 using ELISA. The in vivo function of inductive regulatory T cells was mainly indicated by the expression of IFN-γ, TGF-β1, etc. Frequencyof natural regulatory T cells and inductive regulatory T cells in the peripheral blood samples from Group 1 patients were all significantly higher (P<0.05) than those from Groups 2 and 3.

**Conclusion/Significance:**

Our results indicate that frequency of natural regulatory T cells and inductive regulatory T cells are significantly higher in the peripheral blood of patients with active pulmonary tuberculosis. These findings have potential application in improving TB diagnostic methods.

## Introduction

Tuberculosis (TB) is a disease caused by the chronic and continuous infection of the pathogen *Mycobacterium tuberculosis* (*M. tuberculosis*) [1]. *M. tuberculosis* is an intracellular bacterial pathogen and is eliminated mainly through the CD4^+^ effector Th-cell-mediated cell immune response. These cells, which secrete immunocompetent cytokines, such as interferon (IFN)-γ, play an undeniable role in clearing the bacteria. As the most important immunological characteristics of active pulmonary tuberculosis the immune response of the specific Th1 lymphocytes is suppressed. Many studies have indicated that *M. tuberculosis* can induce regulatory T lymphocytes (Tregs) [4], [5], [6]. As functional T lymphocytes, Tregs mature in the thymus and account for 5%–10% of the total CD4^+^ T lymphocytes in the peripheral blood. They mediate immune suppression, and include two types: natural Tregs (n Tregs), also known as thymus-derived CD4^+^CD25^+^ Tregs, and inductive Tregs (I Tregs). The most important attribute of n Tregs is their ability to inhibit the proliferation of CD4^+^ effector Th cells via direct contact. I Tregs are induced from naive T lymphocytes and have the regulating ability [1] and restrain the proliferation of effector T cells by secreting cytokines such as transforming growth factor-β (TGF-β) and IL-10, and inhibiting the function of immuno-active cytokines.

## Materials and Methods

### Ethics Statement

Approval for this study was obtained from the Ethics Committee of the National Institute for Communicable Disease Control and Prevention, Chinese Center for Disease Control and Prevention. All patients involved in the study provided written informed consent.

### Specimen Collection

We collected blood samples from 3 different populations. The 30 active pulmonary TB patients in Group 1 (17 males, 13 females, average age: 36.7 years old (19 to 72 years old)) were all patients hospitalized from August, 2011 to January, 2012 in the Department of Infectious Diseases, the Heping Hospital of Changzhi Medical College, the People's Hospital of Changzhi City, which were clinically diagnosed with active pulmonary TB based on positive AFB sputum smears and/or cultures, clinical symptoms and chest X-rays (CXR) showing tuberculose signs. The 10 recovered TB patients in Group 2 (7 males, 3 females, average age: 43.6 years old (32 to 49 years old)) were diagnosed by clinical and laboratory examination to have recovered from TB after administration of the standard therapeutic schedule (2HRZE/4HR, H: isoniazid, R: rifampicin, Z: pyrazinamide, E: ethambutol) [2]. The recovered TB patients in this study had completed the treatment and did not have persistent CXR abnormalities. The 30 control subjects (15 males, 15 females, average age: 35.3 years old (19 to 67 years old)) in Group 3 were confirmed as healthy individuals without TB or other infectious diseases using chest X-rays and the conventional PPD skin test. Heparin anticoagulation venous blood samples (2 ml) were collected from participants in the morning after overnight fasting. All the participants in our study were not infected with HIV.

### Reagents and Instruments

Fluorescent antibodies for FITC-CD4, PE-CY5-CD25, PE-Foxp3, and corresponding isotype control antibodies including FITC IgG1 isotype control, PE-CY5 IgG1 isotype control, PE IgG1 isotype control antibodies, and cell lysis solution were purchased from BD (New Jersey, USA). IFN-γ and TGF-β1 kits were purchased from eBioscience (USA). Flow cytometry was performed on a Beckman Coulter flow cytometer, equipped with Expo 32 software.

### Sputum Smears

Fresh sputum samples were collected from patients, smeared and stained by acid-fast staining, and then checked for the presence of acid-fast bacilli under a light microscope, according to “The Laboratory Science Procedure of Diagnostic Bacteriology in Tuberculosis” [3].

### Flow Cytometry

100 µl heparin anticoagulant blood samples were used. 10 µl fluorescein isothiocyanate (FITC)-labeled CD4 and 10 µl phycoerythrin-green pigment (PE-CY5)-labeled CD25 antibody were added for cell surface staining. 1 ml cell lysis solution (1×) was used to lyse erythrocytes, then 250 µl Fix/Perm solution was added to cleave leukocytes. 10 µl phycoerythrin (PE)-labeled Foxp3 was used for intracellular staining. 800 µl flow staining buffer solution was added before analyzing the samples by flow cytometry.

### Determination of IFN-γ and TGF-β1 Levels

Human IFN-gamma platinum ELISA and Human TGF-beta1 platinum ELISA kits obtained from eBioscience (USA) were used according to the manufacturer's instructions to measure serum IFN-γ and TGF-β1 levels of cytokines. A coloured product was formed in proportion to the amount of human TGF-b1 present in the sample or standard. The reaction was terminated by addition of acid and absorbance was measured at 450 nm.

### Statistical Analysis

Statistical analysis was performed using SPSS 18.0 software. Results are presented as means ± standard deviation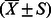. Comparisons between two groups were made using independent sample t-tests. Comparisons between multiple groups were analyzed by one-way Analysis of Variance (ANOVA), and comparisons of two groups among multiple groups were made using LSD t-tests. Differences were considered significant if P<0.05.

## Results

### Flow Cytometry Analysis

**Figure 1:**
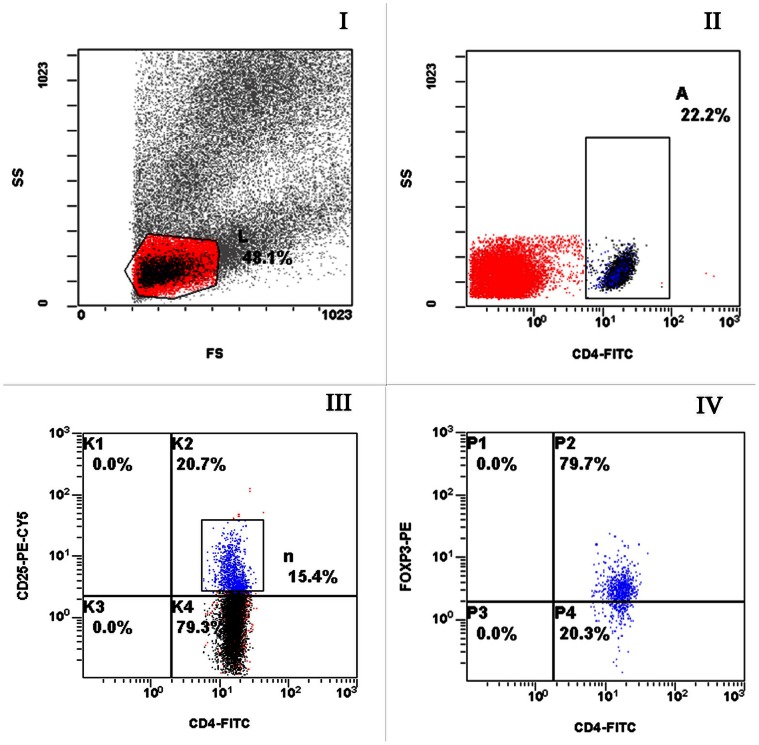
Representative flow plots from an active TB patient labeled with anti-CD4, CD25 and Foxp3. Appropriate isotype controls were included to establish the positive gates. (I) FSC and SSC to identify lymphocytes. (II) CD4^+^ cells within the lymphocyte gate. (III) Cells were gated on the CD4^+^ cells shown in B and CD4^+^CD25^+^ cells were determined. (IV) Cells were gated on CD4^+^CD25^+^ cells and Foxp3 expression was determined.

**Figure 2:**
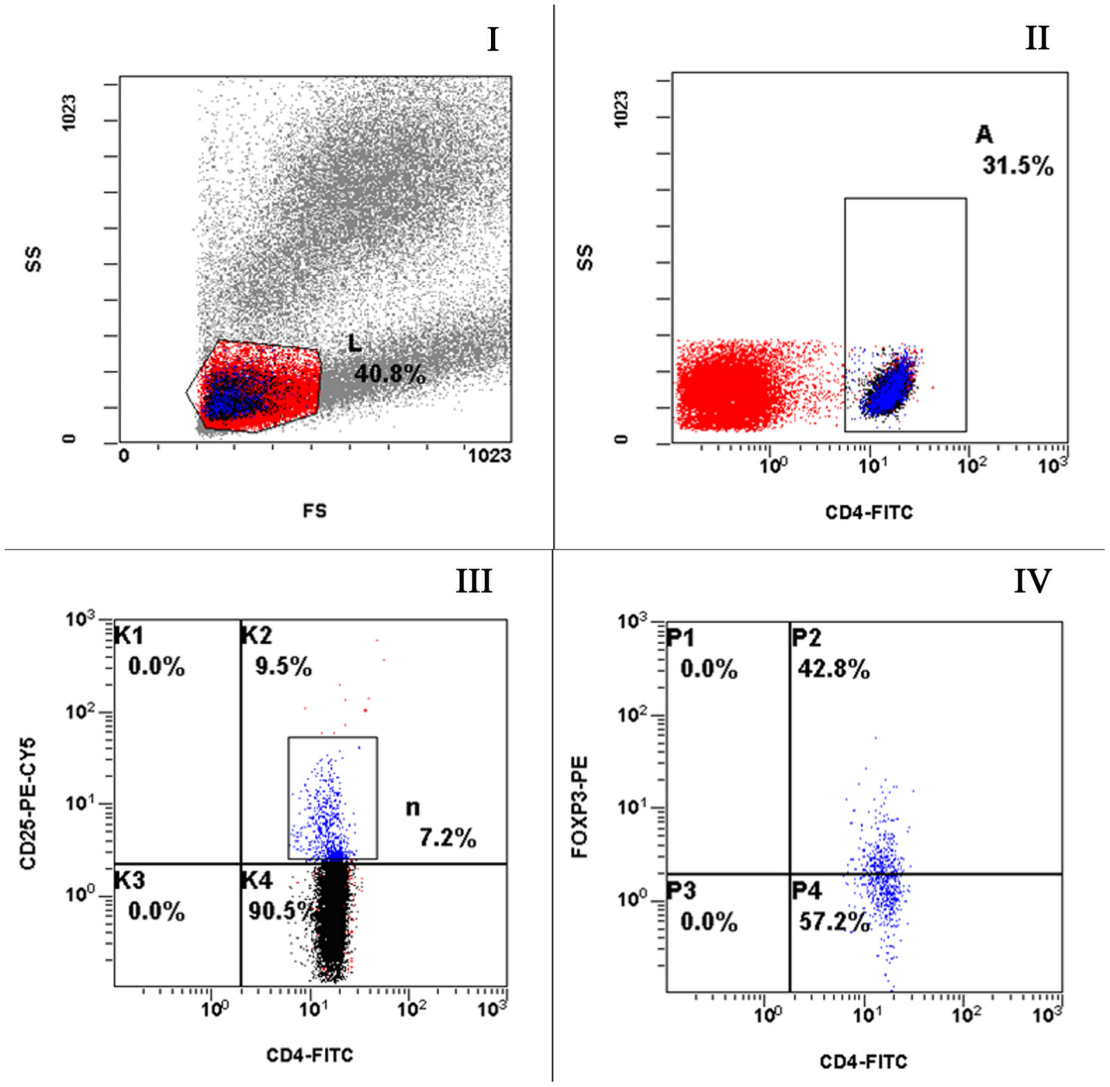
Representative flow plots from a recovered TB patient labeled with anti-CD4, CD25 and Foxp3. Appropriate isotype controls were included to establish the positive gates. (I) FSC and SSC to identify lymphocytes. (II) CD4^+^ cells within the lymphocyte gate. (III) Cells were gated on the CD4^+^ cells shown in B and CD4^+^CD25^+^ cells were determined. (IV) Cells were gated on CD4^+^CD25^+^ cells and Foxp3 expression was determined.

**Figure 3:**
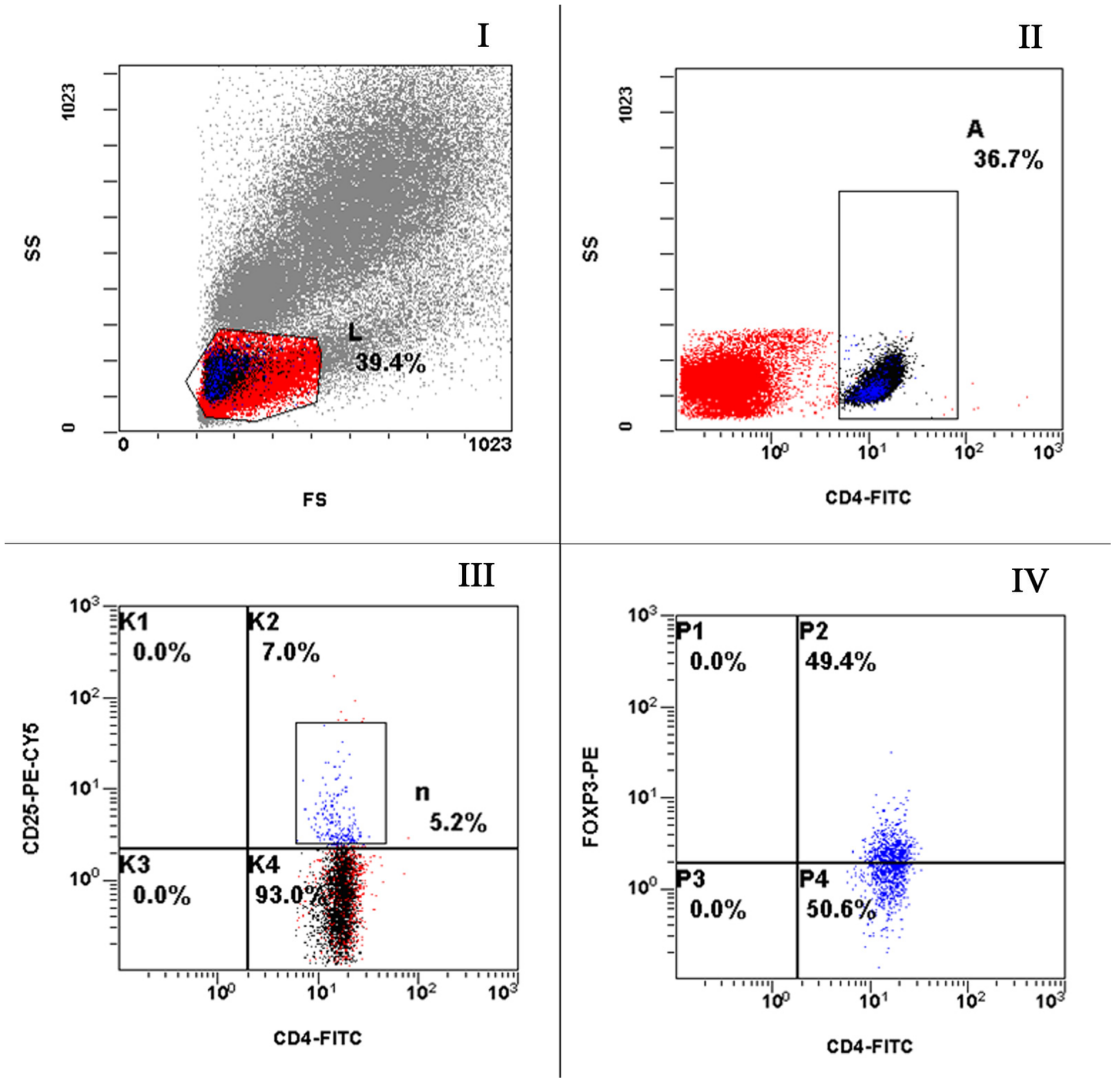
Representative flow plots from a healthy control labeled with anti-CD4, CD25 and Foxp3. Appropriate isotype controls were included to establish the positive gates. (I) FSC and SSC to identify lymphocytes. (II) CD4^+^ cells within the lymphocyte gate. (III) Cells were gated on the CD4^+^ cells shown in B and CD4^+^CD25^+^ cells were determined. (IV) Cells were gated on CD4^+^CD25^+^ cells and Foxp3 expression was determined.

Flow cytometry was used to detect CD4^+^CD25^+^ lymphocytes and CD4^+^CD25^+^Foxp3^+^ lymphocytes. Results for Group 1, Group 2 and Group 3 are shown in Figures 1, 2, 3, respectively; in the figures, L, A, n and P2 representing lymphocytes, CD4^+^ cells, CD4^+^CD25^+^ cells and CD4^+^CD25^+^Foxp3^+^ cells, respectively.

### Regulatory T cells and Related Cytokine Expression

Percentages of regulatory T cells and related cytokine expression in Group 1, Group 2 and Group 3 are shown in Table 1. Results show that the percentages of CD4^+^CD25^+^ cells, and CD4^+^CD25^+^Foxp3^+^ cells were significantly higher in Group 1 than in Group 2 and Group 3 (P<0.05). Levels of TGF-β1 were also significantly higher, but levels of IFN-γ were significantly lower in Group 1 than in Groups 2 and 3 (P<0.05) (Table 1).

**Table 1:** Regulatory T cells and the expression of related cytokines in different patient groups.

Group	Numbers of patients	CD4^+^CD25^+^/CD4^+^ (%)^1^	CD4^+^CD25^+^Foxp3^+^/CD4^+^ (%)^2^	IFN-γ (pg/ml)^ 3^	TGF-β1(pg/ml)^ 4^
Group 1	30	10.41±2.28	7.83±1.96	10.82±6.60	107.10±55.33
Group 2	10	7.04±0.71	4.49±1.22	22.82±2.42	24.13±3.40
Group 3	30	7.45±1.98	4.68±0.92	24.97±2.33	13.89±4.76

1. Group 1 was compared with either Group 2 or Group 3, t  = 4.57, P<0.05 and t  = 4.89, P<0.05, respectively;

2. t  = 5.05, P<0.05 and t  = 7.72, P<0.05;

3. t  = 8.41, P<0.05 and t  = 11.08, P<0.05;

4. t  = 4.70, P<0.05 and t  = 9.18, P<0.05.

### Differences in Regulatory T cells and Related Cytokine Expression in Sputum Smear Negative and Positive Patients

Among the 30 TB patients analyzed, 9 were sputum smear negative and 21 were sputum smear positive. Results showed that percentages of CD4^+^CD25^+^, and CD4^+^CD25^+^Foxp3^+^ cells and the levels of TGF-β1 in sputum smear positive patients were significantly higher than those of sputum smear negative patients (P<0.05), but levels of IFN-γ were lower (Table 2).

**Table 2:** Differences in regulatory T cells and expression of related cytokines in sputum smear negative and positive patients.

Groups		Numbers of patients	CD4^+^CD25^+^/CD4^+^(%)^1^	CD4^+^CD25^+^Foxp3^+^/CD4^+^ (%)^2^	IFN-γ (pg/ml)^3^	TGF-β1 (pg/ml)^4^
Sputum smear (+)	21		11.04±2.33	8.48±1.86	8.34±4.31	127.54±53.25
Sputum smear (−)	9		8.94±1.31	6.31±1.23	16.61±7.58	59.40±18.79

1. Sputum smear positive patients were compared with smear negative patients, t  = 2.51, P<0.05;

2. t  = 3.19, P<0.05;

3. t  = 3.07, P<0.05;

4. t  = 5.16, P<0.05.

### Differences in Regulatory T cells and Related Cytokine Expression in Cavitary TB and Non-cavitary TB Patients

Regulatory T cells and related cytokine expression were analyzed in cavitary TB and non-cavitary TB patients. Of the 30 tuberculosis patients analysed, 19 had cavitary TB and 11 had non-cavitary TB. Results showed that percentages of CD4^+^CD25^+^ cells, and CD4^+^CD25^+^Foxp3^+^ cells, and levels of TGF-β1 were significantly higher in cavitary TB patients than in non-cavitary TB patients (P<0.05), but levels of IFN-γ were lower (Table 3).

**Table 3:** Differences in regulatory T cells and expression of related cytokines in cavitary TB and non-cavitary TB patients.

Groups	Numbers of patients	CD4^+^CD25^+^/CD4^+^(%)^1^	CD4^+^CD25^+^Foxp3^+^/CD4^+^(%)^2^	IFN-γ (pg/ml)^3^	TGF-β1 (pg/ml)^4^
Cavitory TB	19	11.25±1.97	8.61±1.59	6.74±2.89	127.91±55.57
Non-cavitory TB	11	8.96±2.10	6.48±1.84	17.87±4.98	71.15±32.81

1. The cavitary TB group was compared with the non-cavitary TB group, t  = 2.99, P<0.05;

2. t  = 3.33, P<0.05;

3. t  = 6.78, P<0.05;

4. t  = 3.52, P<0.05.

### The Analysis on the Percentage of Lymphocyte/White Blood Cells (%), CD4+ cells/Lymphocytes (%) and CD4+Foxp3+ cells/CD4+ cells

The mean ± standard deviation and the standard error of the percentage of lymphocytes/white blood cells, CD4+ cells/lymphocytes and CD4+Foxp3+ cells/CD4+ cells in the three groups are shown in Table 4.

**Table 4:** The mean ± standard deviation and the standard error of the percentage in different groups.

Groups	Lymphocytes/White blood cells (%)		CD4^+^cells/Lymphocytes (%)		CD4^+^Foxp3^+^cells/CD4^+^ cells (%)	
	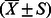	standard error	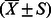	standard error	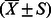	standard error
Group 1	44.33±8.71	1.59	20.83±6.43	1.17	60.46±4.10	0.75
Group 2	41.05±2.05	0.65	31.52±2.08	0.66	46.42±2.73	0.86
Group 3	40.45±6.16	1.12	30.41±5.10	0.93	45.55±3.70	0.68

## Discussion

We have shown that percentages of CD4^+^CD25^+^Foxp3^+^ Tregs in peripheral blood and expression levels of TGF-β1 in active pulmonary tuberculosis patients are significantly higher than those in healthy individuals and recovered patients, while the expression of IFN-γ in active pulmonary tuberculosis patients is lower than that in healthy individuals and recovered patients. This is the first study of T cells in peripheral blood that has been conducted on pulmonary TB patients from Shanxi province and our results are generally consistent with previous reports from other regions [4], [5], [6].

Host immune response against *M. tuberculosis* is mediated by cellular immunity, cytokines and Th1 cells playing critical roles in the process of controlling infection. In particular, protective immunity against *M. tuberculosis* involves the IFN-γ biased Th-1 effector immune response [7], [8], which lead to the varied clinical manifestations of human tuberculosis [9]. During active tuberculosis, suppression of *M. tuberculosis*-specific T cell responses is evidenced by decreased production of cytokines such as IFN-γ [10], [11], [12], with simultaneous overproduction of suppressive cytokines such as TGF-β [13]. The IFN-γ biased Th1 effector response is critical for immune containment of tuberculosis [14]. Several recent reports have demonstrated the presence of the Th-1 like response in the localized form of tuberculosis while its deficit is tightly correlated with disseminated disease [9], [15]. IFN-γ is the main cytokine involved in the immune response against mycobacteria, and its major function is the activation of macrophages, allowing them to exert their microbicidal functions [16].

Natural Tregs (n Tregs) express intracellular forkhead winged-helix family transcriptional repressor p3 (FoxP3), currently described as the most specific marker of Tregs. Expression of FoxP3 correlates well with regulatory activity, and FoxP3 is considered to be a key player in the development and function of n Tregs [17]. FoxP3 represses IL-2, IL-4 and IFN-γ expression and interacts with the nuclear transcription factors of activated T cells (nuclear factor-κB), resulting in poor cytokine production and impaired proliferation [18]. TGF-β is a cytokine that is capable of inducing the expression of Foxp3 in naive T lymphocytes [19]. n Tregs play an important role in the inhibition of CD4^+^ effector T cell expansion and affect CD4^+^ T lymphocytes via direct cell-to-cell contacts. Inductive Tregs (i Tregs) produce high levels of TGF-β1 [20], [21].Activity of Tregs can be reflected through detecting the expression level of TGF-β1. It has been shown that several factors such as IL-2, IL-10 and TGF-β1, which protect host tissues by limiting excess inflammation but may conversely limit the clearance of pathogens [22], are involved in the generation and maintenance of Tregs and can inhibit effector T cell proliferation [23]. i Tregs can also suppress the immune activity of cytokines IFN-γ, and IL-12. In pulmonary tuberculosis patients, CD4^+^CD25^+^Foxp3^+^Treg and TGF- β1 increase, demonstrating that the immune status in these patients is low, and that the pathogen, *M. tuberculosis*, cannot be effectively eradicated. In summary, Tregs play a significant role in the chronicity and persistence of tuberculosis. Comparing and analysing these results in the study showed that levels of CD4^+^CD25^+^/CD4^+^,CD4^+^CD25^+^Foxp3^+^/CD4^+^ and TGF-β1 in pulmonary TB were higher than those in recovered TB patients and healthy controls while IFN-γ were lower. Such conclusion can be drawn that after infecting *M. tuberculosis*, Tregs in patients present high expression, TGF-β1’s level increases while IFN-γ descends due to the restrain of Th-1-mediated cellular immunity simultaneously.

It is well known that most sputum smear positive patients are true TB patients [24]. The amount of *M. tuberculosis* in these sputum smear positive patients is higher thanthose in sputum smear negative patients, accordingly, the activity of Treg cells can be reflected through the percentages of CD4^+^CD25^+^ and CD4^+^CD25^+^Foxp3^+^ cells and the expression of IFN-γ and TGF-β. In our research, the above results of the sputum smear positive patients have shown the same as previous report [25]. Meanwhile, the cavity surface has been described as an area of “failed immunity” [26], [27]. On the inner surface of cavities, there are much more bacteria than other parts of the body. In addition, in a previous study, the percentage of Treg cells was found to increase significantly in the cavitary wall compared to other tissues infected by TB [28]. Therefore, the activity of Treg cells in the cavitary TB patients is stronger than non-cavitary TB patients, and results in our research are consistent with previous study.

### Conclusions

Our results indicate that frequency of natural regulatory T cells and inductive regulatory T cells are significantly higher in the peripheral blood of patients with active pulmonary tuberculosis. These findings have potential application in improving TB diagnostic methods, and we suggest that immunological methods can provide therapeutic value for tuberculosis patients to some extent.
